# From Synthesis to Utilization: The Ins and Outs of Mitochondrial Heme

**DOI:** 10.3390/cells9030579

**Published:** 2020-02-29

**Authors:** Samantha A. Swenson, Courtney M. Moore, Jason R. Marcero, Amy E. Medlock, Amit R. Reddi, Oleh Khalimonchuk

**Affiliations:** 1 Department of Biochemistry, University of Nebraska, Lincoln, NE 68588, USA; sswenson@huskers.unl.edu; 2 School of Chemistry and Biochemistry, Georgia Institute of Technology, Atlanta, GA 30332, USA; cmoore98@gatech.edu; 3 Department of Biochemistry and Molecular Biology, University of Georgia, Athens, GA 30602, USA; Jmarcero@uga.edu; 4 Augusta University/University of Georgia Medical Partnership, Athens, GA 30602, USA; 5 Parker Petit Institute for Bioengineering and Biosciences, Georgia Institute of Technology, Atlanta, GA 30332, USA; 6 Nebraska Redox Biology Center, University of Nebraska, Lincoln, NE 68588, USA; 7 Fred and Pamela Buffett Cancer Center, Omaha, NE 68105, USA

**Keywords:** mitochondria, heme, porphyrin, heme biosynthesis, membrane transporters, hemoproteins

## Abstract

Heme is a ubiquitous and essential iron containing metallo-organic cofactor required for virtually all aerobic life. Heme synthesis is initiated and completed in mitochondria, followed by certain covalent modifications and/or its delivery to apo-hemoproteins residing throughout the cell. While the biochemical aspects of heme biosynthetic reactions are well understood, the trafficking of newly synthesized heme—a highly reactive and inherently toxic compound—and its subsequent delivery to target proteins remain far from clear. In this review, we summarize current knowledge about heme biosynthesis and trafficking within and outside of the mitochondria.

## 1. Introduction

**Figure 1:**
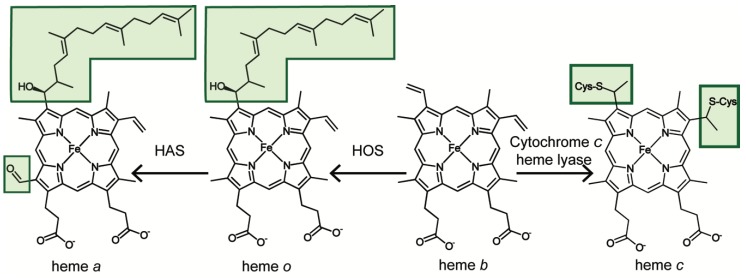
Chemical structures of the various heme types in eukaryotes. Starting with the heme *b* precursor (protoheme), heme *c* is made through covalent attachment to the sulfhydryl moieties of either cytochrome *c* or complex III subunit cytochrome *c_1_*; this process is assisted by the cytochrome *c* lyases. Heme *a* is synthesized from heme *b* through the sequential action of heme *o* synthase and heme *a* synthase enzymes, going through the intermediate, heme *o*.

Heme *b*, or iron protoporphyrin IX (Fe-PPIX), is an essential but potentially cytotoxic protein prosthetic group and signaling molecule. The paramount importance of this metallocofactor is highlighted by the plethora of hemoproteins present in virtually every subcellular compartment that fill vital roles within the eukaryotic cell. As a cofactor, heme is essential for mediating gas synthesis, storage and transport; electron transfer; and chemical catalysis [1,2,3,4,5,6]. As a signaling molecule, heme binding to a number of cellular factors including transcription factors, kinases, ion channels and micro RNA processing proteins [5,7,8,9] or its catabolism to the signaling molecule, carbon monoxide (CO), collectively regulate diverse physiological processes that include oxygen sensing, iron homeostasis, the oxidative stress response, mitochondrial respiration and biogenesis, mitophagy, apoptosis, circadian rhythms, cell cycle progression and proliferation [1,7,10,11,12,13,14,15,16,17,18,19]. Another vital role for heme *b* is to act as a precursor for the synthesis of other heme types important for eukaryotic physiology, including hemes *c*, *o* and *a* (Figure 1). 

All of the aforementioned heme-dependent processes require that heme is dynamically mobilized from its site of synthesis on the matrix side of the mitochondrial inner membrane to heme-dependent proteins throughout the cell [2,3]. However, since heme is a hydrophobic and cytotoxic species [20,21], cells are challenged to mitigate heme toxicity by appropriately regulating cellular heme concentration and bioavailability. The concentration of heme is dictated by the relative rates of its synthesis and degradation [2,3]. Despite the fact that all of the proteins involved in the synthesis and degradation of heme have been structurally characterized to atomic resolution and detailed kinetic studies have revealed their mechanisms of action [2,3], our understanding of how the relative rates of heme synthesis are regulated remains far from clear. Furthermore, the bioavailability of heme is governed by its transport and trafficking; yet, the molecules and mechanisms that mediate these processes are poorly understood. 

Herein, we review the “ins and outs” of heme synthesis, trafficking and utilization, both inside and outside the mitochondria. In particular, we will first discuss the biosynthesis of heme and how it may be regulated to affect heme bioavailability. Next, we will discuss the mechanisms underlying how protoheme IX, or heme *b*, is trafficked and converted to alternative heme types, including hemes *c*, *o* and *a* and how it can exit the mitochondria and be mobilized for use in hemoproteins throughout the cell. Finally, we discuss the latest approaches and technologies that can be utilized to characterize heme transport and trafficking mechanisms. Where appropriate, we will highlight open questions regarding heme homeostatic mechanisms. The mobilization of heme for trafficking and signaling, of which little is understood, represents a challenging frontier in heme cell biology. A better understanding of heme transport is essential for treating numerous diseases associated with defects in heme homeostasis, including certain cancers, cardiovascular diseases and neurodegenerative disorders [19,22,23,24,25].

## 2. Heme Biosynthesis

**Figure 2:**
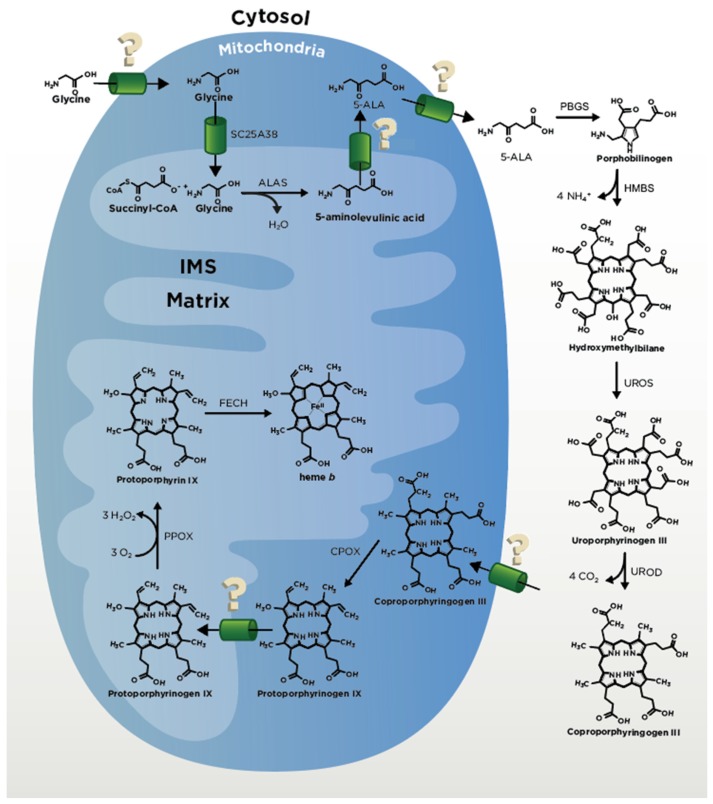
Structures and enzymes involved in heme synthesis shown where they are localized. Glycine is transported into the intermembrane space of the mitochondria (IMS) through an unknown mechanism and is transported across the inner mitochondrial membrane (IMM) by SLC25A38. Succinyl-CoA is synthesized within the matrix. Glycine and Succinyl-CoA are condensed into 5-aminolevulunic acid (5-ALA) by ALA synthase (ALAS). The 5-ALA is transported from the matrix to the cytosol by unknown mechanisms, where Porphobilinogen synthase (PBGS) condensates two molecules of ALA into porphobilinogen (PBG). Four molecules of PBG are combined to form a linear tetrapyrrole hydroxymethylbilane (HMB) by hydroxymethylbilane synthase (HMBS). Uroporphyrinogen III (UPgen III) is synthesized from HMB by uroporphyrinogen synthase (UROS). Coproporphyrinogen III (CPgenIII) is synthesized from UPG III by uroporphyrinogen decarboxylase (UROD). CPgenIII is transported by an unknown mechanism into the IMS. Within the IMS CPgenIII is made into protoporphyrinogen IX (PPgen IX) through catalysis by CPgen oxidase (CPOX). Next, PPgen IX is oxidized into protoporphyrin IX (PPIX) by PPgen oxidase (PPOX). For the final step PPIX is transported into the matrix, where ferrochelatase (FECH) catalyzes the insertion of ferrous iron into the porphyrin ring, resulting in heme *b.*

Cells mitigate the toxicity of heme in part by coordinating heme synthesis (see Table 1) with heme utilization [1]. Animals, fungi and α-proteobacteria make heme via the C_4_ (Shemin) pathway, whereas plants, archaea and other bacteria utilize the C_5_ (glutamate) pathway [26]. Here, we focus on the former, which requires eight enzymes to construct heme from glycine, succinyl-CoA, molecular oxygen (O_2_) and iron (Fe) [12,27,28]. The first and last three enzymes of the heme synthesis pathway in animal and fungi are localized to the mitochondria and the remaining four enzymes reside in the cytosol. Heme synthesis can therefore be controlled at multiple levels, including substrate availability, partitioning of heme synthetic intermediates between the mitochondria and cytosol and direct regulation of the heme biosynthetic enzymes [1,29,30] (Figure 2). In this section we examine each of the enzymes involved in heme synthesis, focusing on their regulation and thus their ability to control heme availability.

### 2.1. ALA Production

The first committed step of heme biosynthesis in non-plant eukaryotes occurs with the pyridoxal 5’-phosphate (PLP)-dependent condensation of glycine and succinyl-CoA to form aminolevulinic acid (ALA) by ALA synthase (ALAS) in the mitochondrial matrix (Figure 2). ALAS is conserved from bacteria to metazoans [31] and is the universal precursor for all tetrapyrroles including chlorophylls, cobalamin, siroheme and coenzyme F_430_ [31,32]. The supply of glycine from the cytosol into the matrix of the mitochondria for ALA production likely occurs by passive diffusion across the highly porous outer mitochondrial membrane (OMM) and then through the inner mitochondrial membrane (IMM) by Solute Carrier (SLC) family 25A38 (Hem25 in yeast) [33,34]. In yeast, one secondary glycine transporter, Ymc1 (another SLC family member), has been determined, but it is currently not known whether this protein has a homolog in humans [35]. The other ALAS substrate, succinyl-CoA, is synthesized in the mitochondrial matrix, primarily from either α-ketoglutarate (α-KG) via α-KG dehydrogenase or succinate via succinyl-CoA synthetase in a cell-specific manner [36,37,38,39].

ALAS structures obtained from α-proteobacterium [40] and yeast [41], both with a high degree of sequence homology to the human ALAS core, indicate a tightly bound homodimer. Specifically, each monomer consists of three domains that all participate in dimerization: a variable N-terminal region, a highly conserved catalytic center with a PLP-lysine Schiff base in the active site and a regulatory C-terminal domain. Eukaryotic ALAS proteins contain a cleavable mitochondrial-targeting sequence (MTS) at the N-terminus and a well-conserved extension of the bacterial C-terminal domain that putatively influences substrate binding [41] and ALA product release [42]. Multiple heme regulatory motifs (HRMs) have been identified in the N-terminal domain of eukaryotic ALAS, including in the MTS, with the notable exception of yeast [43,44,45]. Perhaps not coincidentally, ALA synthesis is the rate-limiting step in mammalian heme synthesis [28] but not in yeast [46]. 

Two highly conserved forms of ALAS, ALAS1 and ALAS2 are expressed in mammals [47]. ALAS1 is ubiquitously expressed, while ALAS2 is specific to erythroid cells [48]. In nonerythroid tissue, feedback inhibition by heme negatively regulates ALAS1 transcription [49], translation [50,51], mRNA stability [52], protein stability in the mitochondria [53] and mitochondrial import via the MTS [54] to avoid free heme accumulation and toxicity. In contrast, ALAS2, which operates in erythroid cells that are obligated to make upwards of ~80 mM heme or ~5 × 10^9^ molecules of heme per cell for hemoglobin assembly [55], is not inhibited by heme at any level. ALAS2 synthesis is modulated transcriptionally by GATA-1 and other heme-independent factors [56,57] and downregulated post-transcriptionally by microRNA [58] and iron regulatory proteins (IRPs) [59]. Interestingly, the inhibitory effect of IRP1 and IRP2 via interaction with an ALAS2-specific 5’-iron regulatory element (IRE) is abrogated by iron and/or heme [60,61], suggesting that heme could indirectly stimulate ALAS2 translation. Unlike in ALAS1, N-terminal HRMs present in ALAS2 protein do not render mitochondrial import sensitive to heme [54]. Post-translational events that impact enzyme activity include the binding of ALAS2 and the AAA+ unfoldase, CLPX, a complex which facilitates both PLP binding and ALAS2 degradation [62], as well as hydroxylation of the ALAS2 C-terminal extension and the associated proteosomal degradation of the protein [63].

### 2.2. CPgenIII Formation

After formation of ALA by ALAS, ALA is transported out of the mitochondria back into the cytosol by unknown mechanisms (Figure 2). Proposed ALA transporters include SLC25A38 [33] and SLC25A39 [64], though these transporters have been implicated in glycine transport [34] and iron-sulfur cluster biogenesis [64] and direct evidence of ALA transport via either is lacking. For transport of ALA from the IMS to the cytosol, it is possible that ALA may simply diffuse across the OMM given the permeability of this membrane to small molecules. Once in the cytosol, ALA is converted in four consecutive steps into CPgen III (Figure 2). These steps include condensation of two ALA molecules into a single monopyrrole, porphobilinogen (PBG), by the enzyme porphobilinogen synthase (PBGS) ((previously known as ALA dehydratase (ALAD)); the head-to-tail synthesis of four PBG molecules to form the linear tetrapyrrole, hydroxymethylbilane (HMB), by the enzyme hydroxymethylbilane synthase (HMBS) (previously called porphobilinogen deaminase (PBGD)); the spiro inversion and cyclization of HMB to form uroporphyrinogen III by uroporphyrinogen synthase (UROS); and the decarboxylation of the four pyrrole acetic acid side chains by uroporphyrinogen decarboxylase (UROD) to yield the final product CPgen III and four molecules of carbon dioxide [12,65]. In contrast to other eukaryotes, PBGS and HMBS (not ALAS) reactions are rate limiting in yeast heme biosynthesis [46].

### 2.3. Coproporphyrinogen Oxidase (CPOX) and Protoporphyrinogen Oxidase (PPOX)

Once generated, CPgen III is then transported by a currently unknown mechanism into the intermembrane space of the mitochondria (IMS) (Figure 2). One study posited that OMM ATP-dependent transporter ABCB6 has a role in this process [66]. However this postulate has been debated based on the cellular localization of ABCB6 [66,67], with biochemical studies which demonstrated binding and transport of porphyrins and not porphyrinogens [66,68,69] and findings from animal studies [68,69]. Thus, additional studies are necessary to further investigate the role of ABCB6 as a CPgen III transporter in heme synthesis. Once CPgen III is in the IMS, the enzyme coproporphyrinogen oxidase (CPOX) converts it to PPgen IX followed by conversion to protoporphyrin IX (PPIX) by protoporphyrinogen oxidase (PPOX), a reaction that requires three molecules of oxygen and generates three molecules of hydrogen peroxide (H_2_O_2_) [12]. A recent study employing proximity labeling, mass spectrometry and electron microscopy showed that PPOX localizes to the matrix-facing side of IMM [70]. These data suggest that PPgen IX rather than PPIX traverses the IMM (Figure 2). The N-terminal moiety of CPOX is anchored in the IMM, so the direct transfer via lipid bilayer is one possible scenario. Alternatively, the transfer could involve a transporter. It was shown that in yeast, heme and some of its precursors may associate specifically with the adenine nucleotide translocator (ANT) and that this binding is inhibited by the ANT substrate ADP [71]. Additionally, Azuma et al. [71] found that disruption of the ANT genes in yeast (*AAC1*, *PET9* and *AAC3*) resulted in a reduction of heme biosynthesis via blockage of precursors from entering the matrix. These transporters may therefore play certain role in tetrapyrroles transport to PPOX and/or FECH. These findings also suggest that ATP:ADP ratios, mitochondrial protonmotive force and cellular energy status may regulate heme synthesis in response to changes in metabolic demand thus further studies are warranted to elucidate this issue. Another recently identified protein which may function in PPgen IX transport is the protein TMEM14C. This protein was originally identified in a large-scale gene expression analysis [64] and shown to be important for erythropoiesis in zebrafish. Yien et al., [72] expanded these studies and their data demonstrate that TMEM14C is important for porphyrin precursor homeostasis thus supporting TMEM14C as a PPgen IX transporter.

### 2.4. Ferrochelatase

The final step of heme synthesis involves the insertion of ferrous iron into the PP IX molecule to produce protoheme by the enzyme ferrochelatase (FECH or Hem15) on the matrix side of the inner membrane (Figure 2). Iron appears to be delivered to FECH via the transporters mitoferrin 1 (MFN1 or SLC25A37) and mitoferrin 2 (MFN2 or SLC25A28) in mammals [73]. A number of recent outstanding reviews focusing on iron delivery to mitochondria can be found elsewhere [74,75,76,77]. FECH functions by removing two protons from PP IX and inserting Fe^2+^. Mammalian FECH functions as a homodimer where each subunit contains a [2Fe-2S] cluster [78]. While the [2Fe-2S] cluster is necessary for enzyme activity [79,80], there is no evidence to support its role in catalysis. Work by Shah et al., [81] indicates that it may be serving as a sensor for the redox or membrane potential in the mitochondrial matrix. Thus, redox signals may serve to integrate energy metabolism with control of heme synthesis. 

Recent biochemical and mass spectrometry-based studies have suggested that the heme biosynthetic machinery forms a large complex, termed the mitochondrial heme metabolon [39,82,83,84]. Indeed, FECH was found to be a component of a multimeric assembly that included the first and seventh enzymes in the heme synthesis pathway, ALAS and PPOX, respectively. In addition, α-ketoglutarate dehydrogenase (KDH) and succinyl-CoA synthase (SUCLA2), which synthesize the heme precursor succinyl-CoA, and TMEM14C, a porphyrinogen transporter [39,82], were also observed in the complex. Independent studies have also shown mitoferrin, a mitochondrial iron importer [84], as well as two ATP binding cassette proteins, ABCB7 [85,86] and ABCB10 [84,86] are protein partners of FECH. These results would suggest that substrate channeling and assembly of the heme metabolon complex may be a key regulatory node for heme synthesis. Most intriguingly, the putative heme chaperones progesterone receptor membrane component 1 (PGRMC1), which has been previously proposed to deliver heme to cytochrome P450 enzymes [87,88,89] and progesterone receptor membrane component 2 (PGRMC2), which delivers heme to nuclear heme dependent transcription factors in adipose tissue [90] also interacts with FECH [83]. This would suggest that certain heme acceptor proteins may interact with the heme metabolon in order to provide an outlet valve for heme distribution to other locales. Altogether, FECH may not just be important for heme synthesis, but also for mediating heme trafficking and distribution via its interactions with other factors.

Once synthesized, heme is trafficked to other cellular locations, including the matrix, the IMM, IMS and any number of extra-mitochondrial locales. However, the molecules and mechanisms that mobilize heme from its site of synthesis are not well defined. Recent work supports the role of a number of proteins in this distribution, including glyceraldehyde phosphate dehydrogenase (GAPDH) [91,92], PGRMC1 [83] and PGRMC2 [90]. Further, it is unclear how the demand for heme in different cellular locations regulates the distribution of heme from FECH.

### 2.5. Anemias and Porphyrias

Disorders in heme synthesis can result in pathological conditions in humans. The most common of these are the anemias which result from a decrease in the number of circulating erythrocytes and/or the amount of hemoglobin in erythrocytes. The most common anemia world-wide is iron deficiency anemia in which heme production during erythroid development is limited due to a deficiency of iron in the diet. This is particularly problematic in women of child-bearing age in third-world countries, since frequent pregnancies deplete body iron stores. Second only to iron deficiency are anemias of chronic disease. These anemias result from the body’s inflammatory response to infection or disease. A deficiency in ALAS2, the first enzyme in the pathway in erythroid precursors, results in sideroblastic anemia [93]. Sideroblastic anemia is characterized by iron accumulation in mitochondria and diminished erythroid cell development. In the case of ALAS2 mutations, iron is available, but heme synthesis is limited.

The disorders known as the porphyrias are caused by aberrant heme synthesis and the concomitant buildup of cytotoxic intermediates in hepatic and/or erythroid tissue. These intermediates may produce symptoms of acute neurological dysfunction and/or cutaneous photosensitivity [94]. Mutations have been identified in all enzymes of the heme biosynthetic pathway except ALAS1 (see reference [93] for comprehensive review). Interestingly induction of ALAS1 in liver resulting from elevated heme oxygenase activity [95], consumption of xenobiotics [96] or steroids [97] and poor nutrition [98] can precipitate acute porphyric attacks. The most recently discovered porphyria is X-linked protoporphyria, which results from gain-of-function mutations in ALAS2 [99]. This porphyria resembles erythropoietic protoporphyria, which is caused by decreased FECH activity. This phenotype similarity points to metal chelation by FECH as a second regulatory step in heme synthesis and hints at some level of regulatory coordination between the first and last steps of the pathway.

At present, there are idiopathic anemias and porphyrias for which no underlying molecular cause has been identified. The identification of the multi-protein mitochondrial heme metabolon and other protein partners of heme biosynthesis enzymes opens a black box that in time may shed light on additional means of heme synthesis regulation. Clearly any mutations in these interacting proteins that cause dysfunction in heme synthesis may contribute to porphyrias and anemais. One example is a recently discovered CLPX mutation, which has been implicated in porphyria [62]. Additional studies on the function and interactions among the protein components will further our understanding of the regulation of heme synthesis and, thus, the pathophysiology of the anemias and porphyrias.

## 3. From Heme *b* to Hemes *c*, *o* and *a*

All mitochondrial heme species are generated from the heme *b* produced by FECH (Figure 1). Each of the different heme species is necessary for a specific function and is generated by its own diverging pathway. While much is known regarding heme synthesis, less is known about heme modification and distribution and most available information has been largely elucidated in yeast studies. Since the heme *b* product release by FECH is rate-limiting [100], the interaction between FECH and heme chaperone is likely what regulates and distributes heme throughout the cell [12]. This protein-mediated shuttling of heme is the probable means of distribution, as “free” heme is inherently toxic and can catalyze the formation of harmful reactive oxygen species [12,101]. These predictions are in line with a recent report estimating that less than one molecule of exchange-labile heme is available in the mitochondrial matrix compartment [92].

As noted above, all other species of heme are generated from heme *b*, including hemes *a*, heme *o* and heme *c*. These different species of heme diverge from one another due to various modifications of the porphyrin ring. Heme *c* is the only heme that is covalently bonded to the protein in which it is assembled. The two vinyl (C=C) side chains of heme *c* are covalently attached to the cysteine sulfhydryl residues of the apoprotein (Figure 1). Two examples of heme *c* containing proteins are the cytochromes *c* and *c_1_*. Heme *o* and heme *a* are formed in a two-step consecutive process by the proteins Heme *o* Synthase (Cox10) and Heme *a* Synthase (Cox15), respectively. Heme *o* is modified from heme *b* by the farnesylation of a vinyl group at the C_2_ position of the porphyrin ring [6,102] and heme *a* has the C_8_ methyl substituent oxidized to an aldehyde group on top of this [103,104,105,106] (Figure 1). In all cases, heme *b* must be mobilized from FECH via currently unknown mechanisms to be converted into these other heme types. 

In eukaryotes, heme *o* exists only as long as it takes to convert it to heme *a*. In some bacteria, however, heme *o* can be used as a prosthetic group in some terminal oxidases, such as cytochrome *o*-containing *bo_3_* oxidase—one of two terminal ubiquinol oxidases in *Escherichia coli* [107]. The *bo*_3_ oxidase is both structurally and functionally related to cytochrome *c* oxidase (CcO) of mitochondria and bacteria, except that heme *o* cannot function as a prosthetic group in these other species [108]. Heme *a* is utilized in the active site of Cox1, the central core subunit of CcO, or Complex IV, of the electron transport chain (ETC). CcO harbors two heme *a* molecules—heme *a* and heme *a_3_*—the distinction between the two being their coordination geometries within the protein. Heme *a* is a 6 coordinate, low spin, cofactor and heme *a_3_* is a 5 coordinate, high spin, cofactor, giving two identical molecules with significantly different physical and chemical properties within CcO. This is the only known physiological function of heme *a* [6,108]. In the following sections of this review, we will summarize currently available information regarding mitochondrial hemoproteins and highlight gaps in our knowledge related to the distribution of different heme species to these proteins. 

### 3.1. Mitochondrial Heme b Pathways

**Figure 3:**
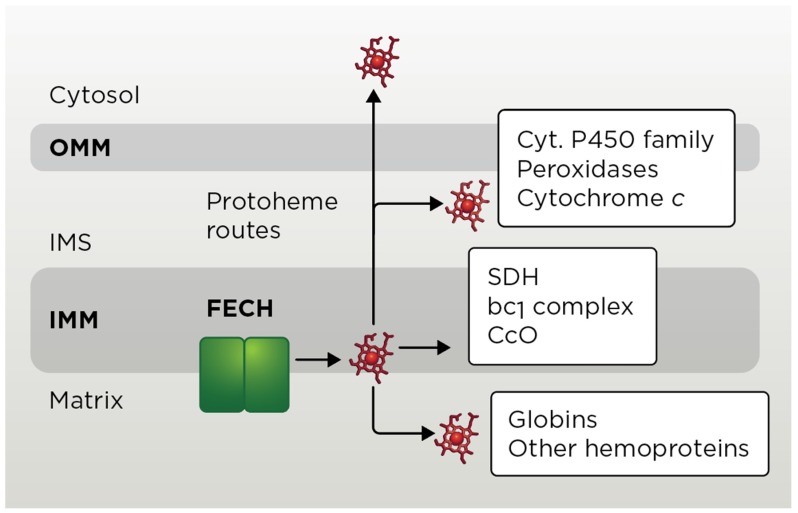
Mitochondrial heme routes. Mitochondrial enzyme ferrochelatase (FECH) metallates PP IX, yielding protoheme that is routed to several suborganellar locales, wherein heme *b* is sequentially modified and/or inserted into the indicated hemoproteins. SDH, succinate dehydrogenase; CcO, cytochrome *c* oxidase; OMM, outer mitochondrial membrane; IMS, intermembrane space; IMM, inner mitochondrial membrane.

Heme *b* (aka Protoheme IX) is utilized as a cofactor by several mitochondrial proteins/complexes (Figure 3). These include mitochondrial matrix-localized flavohemoglobin Yhb1 (neuroglobin in mammals) [6,109], IMM-anchored ETC enzymes ubiquinol-cytochrome c oxidoreductase (complex III, *bc*_1_ complex) [110] and succinate dehydrogenase (complex II) [6,111,112]. Additionally, yeast mitochondria harbor several fungi-specific hemoproteins such as CcO assembly factor Mss51 [113], cytochrome *c* peroxidase Ccp1 [114] and *L*-lactate cytochrome *c* oxidoreductase Cyb2 [115]. Remarkably little is known about how heme *b* is transported to these targets. As exemplified by studies on hemylation of complex III, formation of heme *b* catalytic centers is an early post-translational event in the assembly of these molecules [6,116]. Such setting likely assures an effective heme-dependent regulatory mechanism, thereby reducing potential negative effects of cofactor’s exposure.

### 3.2. Heme c Pathway

Unlike the other heme types, heme *c* needs to be covalently attached to its client proteins—cytochrome *c* (Cyc1) and complex III subunit cytochrome *c*_1_ (Cyt1). In each case, hemylation of these proteins involves the formation of two stereospecific thioether bonds between two cysteine residues of protein’s conserved Cys-X-X-Cys-His motif and two vinyl groups of the protoheme. The histidine residue is an axial ligand to heme iron. The covalent attachment of heme to *c*-type apo-cytochromes is critical for their stability and subsequent maturation and has been extensively studied [6,117,118]. In yeast, hemylation of apo-Cyc1 and apo-Cyt1 is mediated by either of the two IMS-localized cytochrome *c* heme lyases—CCHL (Cyc3) and CC_1_HL (Cyt2) [117,119]. In mammals, a single enzyme, holocytochrome *c* synthase (HCCS), is responsible for the covalent heme attachment. All mitochondrial CCHL proteins appear to use the following *modus operandi*: IMM-bound lyase binds reduced Fe-PPIX, which is followed by binding of apocytochrome *c* or *c*_1_ and subsequent formation of covalent thioether bonds and release of hemylated cytochrome [120]. Although heme binding to CCHL proteins is well characterized [120], how reduced heme is transported to CCHL proteins across IMM remains to be determined. 

In vitro analyses indicate that *c*-heme lyases can directly catalyze the formation of a thioether bond within the CCHL–ferrous heme-apocytochrome *c*/*c*_1_ complex, wherein apocytochrome provides the second histidinyl ligand to this complex with the first axial ligand being supplied by invariant histidine residue of CCHL. However, a scenario whereby CCHL proteins merely serve as stereospecificity-assuring chaperones allowing for spontaneous thioether bond formation is also possible. Likewise, it remains to be determined whether any additional proteins may be facilitating this process in vivo. For instance, studies in yeast identified the IMS-residing flavoprotein Cyc2 that might function as a heme reductase or redox modulator of heme lyase reactions [121,122]. Because Cyc2 is not absolutely required for CCHL function and is not conserved outside fungi, its physiological significance remains somewhat unclear. Nevertheless, these findings suggest that additional factors may be involved in the maturation of *c*-type cytochromes in vivo. 

Once heme is covalently ligated, the resulting holocytochrome *c*/*c*_1_ is released from heme lyase. The release is facilitated by several factors including conformational distortion of the heme molecule upon its covalent biding to cytochrome *c*/*c*_1_ [120,123] and holocytochrome’s axial ligand-mediated dwindling of heme-CCHL adducts [118,120]. Interestingly, the heme lyase’s residues that have been implicated in heme coordination also appear to contribute to its release [120]. Upon its hemylation the stabilized cytochrome *c* is further folded into its functional conformation and cytochrome *c*_1_ is conveyed for further incorporation into maturing *bc*_1_ complex. It will be interesting to test whether hemylation of cytochromes *b* and Cyt1 are coordinated molecular events.

### 3.3. Heme a Pathway

Heme *a* is a modified Fe-PPIX uniquely used by CcO, a key heme-copper enzyme of the ETC [6,124]. Two heme *a* molecules with different coordination geometries—designated *a* and *a*_3_—reside in the Cox1 core subunit of CcO and are essential for catalysis and the stability/folding of Cox1 [6,125]. Hemylation of Cox1 is a sequential post-translational process [6,126,127]. Heme *a* synthesis requires the shuttling of the compound’s precursors—hemes *b* and *o—*between heme *a* biosynthetic factors, and the subsequent shuttling of mature heme *a* to maturing CcO. This process is mediated by a number of different assembly factors [128,129,130,131], but much remains to be elucidated in terms of the molecular mechanisms at work.

#### 3.3.1. Heme *o* Synthase

As discussed earlier, heme *o* synthase, Cox10, is the protein responsible for converting one of the vinyl carbons of the heme *b*’s macrocycle into a hydrophobic hydroxyfarnesyl tail, yielding heme *o*, the precursor for heme *a* (Figure 1) [102]. This evolutionarily conserved 46 kDa-enzyme is intrinsic to the IMM and it is predicted to contain 8 to 9 transmembrane helices that form a catalytic site on the matrix face of the inner membrane. Cox10 assembles in presumably homo-oligomeric complexes of approximately 300 kDa [132,133]. Some structure-function insights into Cox10 can be potentially gleaned from the recently solved structure of UbiA, a distantly related bacterial prenyl transferase [134], but no information exists on how heme *b* is shuttled to Cox10’s active site. Cox10 multimerization seems to be dependent upon the presence of newly synthesized Cox1 and its early assembly intermediates [131,132,133]. The oligomerization, but not steady state levels, of Cox10 is dependent upon the presence of the yeast-specific Cox10 effector protein Coa2 [132,133]. However, the gain-of-function mutation of Cox10, N196K that lies close to matrix side of TMD2 suppresses the Coa2 deletion phenotype [132]. Identification of the N196K substitution led to the conclusion that Cox10 activation may be linked to a multimeric structure. Because Cox10 is present at approximately 8-fold lower abundance than the heme *a* synthase enzyme discussed below, the synthesis of heme *o* is likely the rate-limiting step of heme *a* production [135].

#### 3.3.2. Heme *a* Synthase

Heme *a* synthase, Cox15, catalyzes the conversion of heme *o* to heme *a*. Cox15-mediated conversion of one ring methyl carbon to a formyl group increases the mid-point potential of heme *a* approximately +180 mV, thereby permitting more efficient catalysis by CcO [136,137]. This enzyme is highly conserved from bacteria to humans, allowing for much insight in its function to be garnered from model organisms such as *Saccharomyces cerervisiae*. Cox15 is proposed to have eight transmembrane domains (TMDs) and is anchored in the IMM. Of these eight TMDs, models of Cox15 predict the protein to contain two domains, each comprised of a 4 helix bundle connected by a short (approximately 20 amino acids) unstructured linker region. A very recently solved crystal structure of *Bacillus subtilis* heme *a* synthase homolog confirms these predictions [138]. Removal of the linker region impairs Cox15 function, suggesting its importance in enzyme activity [139]. The sequence similarity between the first domain and the second suggest the possibility of a gene duplication occurring early in its evolutionary history. Cox15 has been found to form higher-order homo-oligomeric protein complexes, on the range of the ~350–450 kDa in size, however the importance of the formation of these complexes on Cox15 enzymatic function remains to be clarified [139]. Of note, modeling of the R217W Leigh syndrome (LS)-associated substitution in yeast revealed the mutation affects oligomeric properties of Cox15 [139]. In contrast to Cox10, the Cox15 active site is predicted to reside on the IMS side of the IMM; no information is available as to how heme *o* is transported across the IM to the Cox15 active site. Another riddle to this is the fact that eukaryotic Cox10 and Cox15 do not stably interact [132] and are differentially regulated [135,139]. Unlike *COX10*, *COX15* gene expression is regulated by the transcription factor Hap1, which is itself regulated by heme concentration [140]. As such, Cox15 protein levels are strictly regulated by heme availability [135,139]. It has been suggested by studies of the bacterial Cox15 that the protein contains a heme *b* cofactor [106], however, whether this proposed cofactor is necessary for enzymatic function or maintaining protein structure has yet to be determined. There are four invariant histidine residues in the protein, two in each of the domains. Mutation of any one of these residues in bacterial species was found to ablate heme *a* [106]. Similarly, in the yeast Cox15, mutations in these invariant histidine residues are critical for the enzyme’s function and cannot be substituted with other heme-ligating amino acids [139]. The structural study by Niwa et al. [138] on *B. subtilis* heme *a* synthase indicates that the N-terminal half domain of Cox15 is likely constitutes heme *o* substrate binding site, whereas the C-terminal domain of the enzyme harbors a heme *b* cofactor molecule. This structure also implicated an ultraconserved glutamate residue (Glu-57 in the bacterial enzyme) as the catalytic residue [138]. However, the exact mechanistic role of these residues in eukaryotic Cox15 remains to be validated.

In *S. cerevisiae*, the Cox15-catalyzed conversion of heme *o* to heme *a* is known to occur in conjunction with the mitochondrial ferredoxin and ferredoxin reductase, yeast adrenodoxin homolog 1 (Yah1) and yeast adrenodoxin reductase homologue 1 (Arh1), respectively [103,104,105,141]. The heme *b* moiety of Cox15 is likely a key cofactor for electrons supplied by this redox couple. Yah1 and Arh1 are mitochondrial [2Fe-2S] proteins that are important for the maturation of Fe/S proteins [142,143]. Yah1 receives its electrons from Arh1, which utilizes NADH as its electron source. These two proteins supply Cox15 with electrons and thus are critical for the formation of heme *a* [103,141]. Of note, in *Schizosaccharomyces pombe* Yah1 and Arh1 exist as portions of a tandem fusion protein [144]. The yeast Yah1 functions as an electron donor for both heme *a* and iron-sulfur cluster biosynthetic machineries, whereas mammalian mitochondria are equipped with two isoforms of ferredoxin—FDX1 and FDX2—where FDX1 functions specifically in steroidogenesis and FDX2 is specific for heme *a* and iron-sulfur cluster biogenesis [145].

#### 3.3.3. Other Proteins Related to Heme a Biogenesis

Additional factors have been implicated in maturation and/or transport of heme *a* to its final destination. This group of proteins includes CcO assembly factors Pet117, Shy1/SURF1 and the yeast-specific protein Coa2. 

Pet117 was identified as a conserved 107-amino acid-long C*c*O assembly factor by McEwen et al. [146]. Since its discovery, little work has been done to characterize the protein. Deletion of the *PET117* gene was shown to have a reduction in heme *a* levels [147], indicating that Pet117 may function in heme *a* biosynthesis in some manner. We recently showed that Pet117 is the mitochondrial protein localized to the matrix side of the IMM [148]. More importantly, we discovered that Pet117 stably interacts with Cox15; this interaction is independent of catalytic function of Cox15, but deletion of the 20-amino acid unstructured linker region of Cox15 abolishes the interaction. While Pet117 does not appear to be a heme-binding protein per se, its deletion results in a loss of Cox15 oligomerization, indicating that Pet117 promotes stabilization of Cox15 oligomers, thus facilitating heme *a* biosynthesis. Based on the observation that the Pet117-Cox15 interaction is ablated in mitochondria lacking Cox1 transcriptional activator Mss51 [148], it is tempting to speculate that Pet117 may be coupling Cox15 oligomers to maturing Cox1 assembly intermediate. 

Shy1/SURF1 is a conserved IM-anchored CcO assembly factor whose exact function has yet to be clarified. In both yeast and mammals, newly synthesized Cox1 progresses through a series of intermediates containing different assembly factors. Formation of the heme *a*_3_:Cu_B_ bimetallic center appears to occur in an intermediate containing Shy1 [126,131,149]. It remains unclear, which Cox1 assembly intermediate triggers Cox10 and Cox15 activation and oligomerization [133]. Recent reports indicate that Shy1 substoichiometrically associates with Cox15, so Shy1 is likely to play a certain role in CcO hemylation [149]. Studies with recombinant *Pseudomonas denitrificans* SURF1 isoforms shown the protein can stoichiometrically bind heme *a*, leading to the conclusion that this protein can act as a heme chaperone in Cox1 hemylation [150,151]. However, subsequent analyses of Shy1/SURF1 in eukaryotes have challenged this postulate. Shy1 is partially dispensable as null mutants retain a significant percent of CcO activity [152]. In the human Shy1 homologue, SURF1, the same is true for several cases of the truncating SURF1 mutation [153]. In yeast, *SHY1* mutants only produce 10–15% of the fully functional CcO complex, similar to Leigh Syndrome (LS) patients who exhibit 10–30% of normal CcO. Moreover, mutation of conserved candidate heme-ligating residues had little to no effect on the protein’s function indicating that Shy1 is unlikely to be a dedicated heme chaperone [152]. Shy1/SURF1 may thus be simply facilitating CcO hemylation by stabilizing the maturing Cox1 subunit, but further analyses are warranted to validate this model.

Coa2 is an assembly factor for CcO in yeast, originally identified as a high-copy genetic suppressor of the *shy1*Δ mutation [154]. This small (~11 kDa), matrix-localized protein functions downstream of insertion of newly synthesized Cox1 into the IM and it has been shown to physically interact with Shy1 [154]. The newly synthesized Cox1 is rapidly degraded in the absence of Coa2, presumably due to impaired hemylation and misfolding of this CcO subunit [132,154,155]. Just like Shy1/SURF1, Coa2 does not appear to be a bona fide heme chaperone, but its deletion affects oligomerization of both Cox10 [132,133] and Cox15 [133]. Interestingly, our data suggest that unlike Cox10, Cox15 still oligomerizes in *coa2*∆ cells, but that the complex is more labile and collapses under blue native electrophoresis conditions (our unpublished data). This indicates that Coa2 is likely not required for Cox15 oligomerization but plays more of a stabilizing role for Cox15 oligomers, similar to the Pet117 protein. As mentioned previously, the respiratory defect of *coa2*Δ cells is specifically suppressed by the dominant gain-of-function N196K mutation in Cox10 [132] or—to much lesser extent—by the enhanced Cox1 synthesis [133]. Consistent with Coa2’s role in HOS oligomerization, both suppression mechanisms are associated with the abundance of Cox10 oligomer. These observations led to a conclusion that Coa2 may be important for coupling Cox1 synthesis to Cox15 oligomerization and/or activity [133]. An outstanding question remains if such principles can be applied to mammalian heme *a* transport, as it is currently unclear whether a human homolog of Coa2 exists.

### 3.4. Heme c and Heme a Pathway-Related Diseases

Several human diseases stem from defects in heme *c* and heme *a* pathways. The hereditary dominant microphthalmia with linear skin defects (MLS) syndrome-associated mutations in HCCS have been linked to both defective heme binding and cytochrome *c* release, highlighting the importance of these processes to human physiology [156,157]. These findings also strongly suggest that heme binding to CCHL proteins is a rate-limiting step in cytochrome *c* maturation.

Mutations in conserved residues of Cox10, Cox15 and SURF1 manifest in tubulopathy and leukodystrophy [158], sensorineural deafness [159], fatal infantile hypertrophic cardiomyopathy [159,160,161], Charcot-Marie-Tooth disease type 1A [162] and neurologic LS [153,159,163,164,165,166,167,168,169,170]. Of note, pathologic mutations in SURF1 account for the majority of cases of LS associated with CcO deficiency [171].

The yeast Pet117 has a human orthologue that is approximately 81 amino acids in length and that has not been thoroughly studied. One study proposed that human PET117 might be involved in copper insertion to CcO [172]. However, more recent reports have challenged that idea. In addition to our study in yeast, a premature stop codon patient mutation in *PET117* has been identified [173]. This mutation results in a LS-like condition from complex IV deficiency—reduction in steady state levels of complex IV as well as the core subunits, Cox1–3 and reduction in complex IV activity levels. Treatment of patient fibroblasts with exogenous copper was unable to rescue the complex IV deficiency, indicating that PET117 likely does not function as a copper chaperone [173]. The role of PET117 in heme *a* biosynthesis in humans has yet to be clarified.

## 4. Extra-Mitochondrial Heme Trafficking

**Figure 4:**
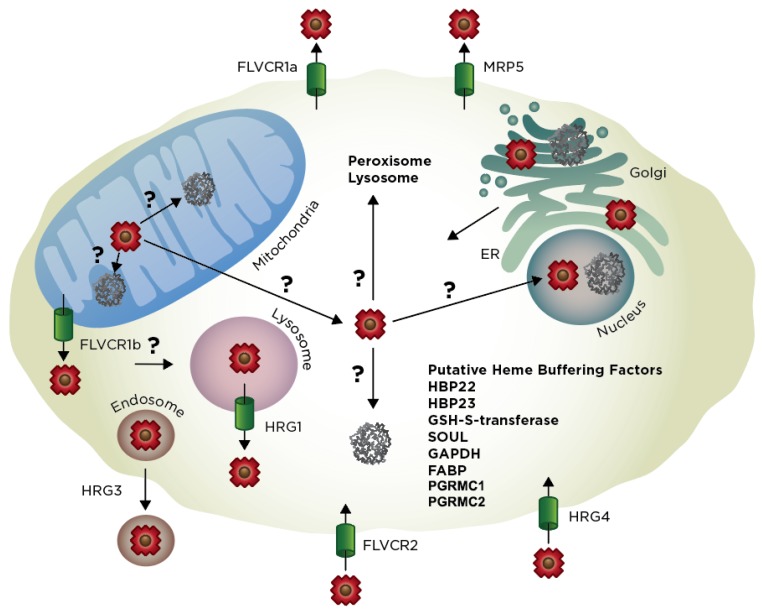
Schematic representation of heme trafficking within a eukaryotic cell. Heme synthesis is completed within the matrix of the mitochondria; from here heme (red polygons) is mobilized throughout the cell to the cytosol, IMS, peroxisome, lysosome, endoplasmic reticulum (ER), Golgi, nucleus and extracellular space. Known heme buffering factors and transporters are highlighted, any unknown pathways are marked with question marks.

Cellular heme is either derived endogenously from de novo synthesis or exogenously from heme uptake (Figure 4) [2,3,174]. Once synthesized by FECH or transported into the cell, heme must be mobilized and delivered to heme-dependent proteins residing in virtually every subcellular compartment. As with other transition metals, e.g., copper or iron, it is generally assumed that heme distribution is mediated by the coordinated action of heme transporters, chaperones and carrier proteins [2,3]. However, unlike other transition metals, heme is also a hydrophobic lipid-like molecule [3]. This raises the possibility that heme could be trafficked like other mitochondrial derived lipids via vesicles or through contact sites between organelles. In this section, we highlight our current understanding of the journey heme takes from its site of synthesis or uptake to locations throughout the cell.

### 4.1. Exit of Mitochondrial Heme

How is heme trafficked out of the mitochondria? One possibility is that it is exported via dedicated heme transporters (Figure 4). The only mitochondrial heme transport protein identified to date is Feline leukemia virus subgroup C receptor-related protein 1b (FLVRC1b), a member of the major facilitator superfamily (MFS) of secondary active transporters [175]. FLVCR1b is a six-TMD protein that is derived from an alternative transcriptional start site located within the first intron of *Flvcr1* [175], which otherwise encodes FLVCR1a, a twelve TMD plasma membrane heme exporter [14,176]. The primary evidence of the role of Flvcr1b in mitochondrial heme export stems from over-expression and depletion studies; silencing of Flvcr1b transcript using siRNA results in mitochondrial heme accumulation and termination of erythroid differentiation while overexpression results in increased cytosolic heme [175]. While the genetic evidence for the role of Flvcr1b in heme export is quite strong, there are a number of unresolved questions. For instance, there is still no direct biophysical evidence that Flvcr1b can transport heme or mechanistic understanding of heme translocation. Interestingly and quite importantly, a segment of Flvcr1a found to be important for heme binding and transport, e.g., residues 132–201 and in particular His-145, Tyr-153 and His-198, are missing in Flvcr1b [177]. Additionally, it is not known whether Flvcr1b is located on the inner or outer mitochondrial membranes, which will have implications for where it acquires and transports heme, i.e., matrix to IMS vs. IMS to cytosol. In the context of the heme metabolon, it is tempting to speculate that Flvcr1b could interact with FECH or other components of the heme biosynthetic machinery and accept heme for transport immediately upon its synthesis. However, data from affinity purification studies of FECH do not support this interaction [82].

Affinity purification data do support interactions between FECH, PGRMC1 and PGRMC2 [83]. Both PGRMC1 and PGRMC2 have been proposed to be heme chaperones [90,178,179]. As mentioned earlier PGRMC1 and PGRMC2 are both proteins that bind heme with moderate affinity and either stimulate hemoproteins including cytochrome P450s [87,88,89] or alter labile heme levels in different cellular compartments [90]. Interestingly PGRMC1 has been localized to multiple cellular locations including the ER [180], mitochondria [83], nucleus [181] and cellular membrane [182], while PGRMC2 is an ER protein [90]. Multiple studies have found that PGRMC1 and PGRMC2 interact [90] and recent studies using adipose-tissue specific knock out of PGRMC2 or both PGRMC2 and PGRMC1 result in nuclear heme homeostasis disruption and metabolic dysfunction [3]. Thus, PGRMC1 and PGRMC2 have been proposed to function as a conduit for heme transport from FECH in the mitochondrial matrix to the ER and nucleus [90]. Further studies of these proteins will shed light of the mechanisms by which these proteins function in cellular heme trafficking.

Instead of heme export from the mitochondria via a single transport protein, there are likely multiple routes of heme export. Indeed, *S. cerevisiae* does not encode Flvcr1 homologs and has a single PGRMC1 and PGRMC2 protein (Dap1), implying the presence of alternative heme transport mechanisms (Figure 4). In addition to other yet to be determined heme transporters, alternative pathways for mobilizing mitochondrial heme involves its passage through membrane contact points or vesicular trafficking to other organelles [3]. With respect to the former, there may be direct heme transfer between the mitochondrial and ER networks via mitochondria-associated membranes (MAMs). MAMs form a contiguous interface between the mitochondrial outer-membrane and the endoplasmic reticulum (ER) via the ER-mitochondria encounter structure (ERMES). While ERMES has previously been implicated in facilitating the exchange of lipids between the ER and mitochondria, it may also serve as a route for heme transfer as well [183]. While direct evidence for the role of MAMs and ERMES in facilitating heme exchange is lacking, it is worth noting that a number of heme homeostatic factors, including CPOX, FECH, heme-binding protein 1 (HBP 1), heme oxygenase-2 (HO-2) and PGRMC1 have been found to associate with MAMs [184,185]. Since the ER network extends between the nucleus, Golgi and secretory pathway, once heme exchanges into the ER, it can bind to any number of hemoproteins present in these locations, as well as proteins that are eventually secreted or are directed to the lysosome, peroxisome, or plasma membrane. However, the mechanisms underlying heme distribution in the ER or Golgi are currently unknown. Another potential mechanism for the export of mitochondrial heme is via mitochondrial-derived vesicles (MDVs) [186]. MDV’s have been found to traffic material to peroxisomes and lysosomes and it is possible that heme may also be packaged in these vesicles as cargo. 

### 4.2. Import of Exogenous Heme

Heme can be acquired from exogenous sources in addition to de novo synthesis. As with mitochondrial heme export, heme import could be mediated by a number of different mechanisms, including transporters, hemoprotein receptors, endocytic pathways or lipid carriers (Figure 4). The first *bona fide* eukaryotic heme transporters, HRG-1 and HRG-4, were identified using molecular genetic approaches in *Caenorhabditis elegans*. As a heme auxotroph, all heme in *C. elegans* is derived from nutritional sources and is mobilized from the intestines to tissue throughout the animal [187]. Heme responsive genes HRG1 and HRG4 were first identified from a transcriptomic screen as genes that are up-regulated during growth on low heme and were later determined to be involved in intestinal heme uptake in worms [188]. HRG-4, which lacks mammalian homologs, localizes to the plasma membrane, while HRG-1, which is conserved from arthropods to vertebrates, including mammals (20% identity between human and *C. elegans* HRG1), is present in endosomes and transports exogenously-derived heme into the cytosol.

Human HRG1 is predicted to have four TMD-spanning helices [188,189] and functions cooperatively with a V-type H^+^-ATPase. Indeed, acidification of endosomes is necessary to protonate heme coordinating residues of hemoproteins to facilitate the release of heme, solubilize unbound “free” heme and couple heme export out of endosomes to a proton gradient [189]. A number of lines of evidence have implicated HRG1 as a heme transporter, including (i) HRG-1-mediated import of heme into *Xenopus* oocytes; (ii) HRG-1-mediated transport of zinc mesoporphyrin (ZnMP) into the *C. elegans* intestine and ectopically expressing murine cells; and (iii) HRG-1 heme transport activity using yeast reporter assays. Furthermore, a number of highly conserved heme-binding His and Tyr residues are critical for HRG1 activity [190]. Functionally, HRG-1 is critical for mobilization of heme from the phagolysosome of macrophages during erythrophagocytosis [191] and heme uptake and utilization in the *C. elegans* intestine [188].

An additional putative cell surface heme transporter is FLVCR2 [192]. Indeed, FLVCR2 binds to hemin-agarose, FLVCR2 overexpression in Chinese hamster ovary (CHO) cells and *Xenopus laevis* oocytes leads to accumulation of exogenous heme and enhanced sensitivity to heme toxicity and FLVCR2 depletion by siRNA leads to reduced accumulation of the fluorescent heme analog, zinc mesoporphyrin. On the other hand, unlike HRG-1 and HRG-4, when FLVCR2 is expressed in a yeast system, it cannot transport heme to rescue heme-deficient strains [190]. Furthermore, while there is no clear physiological role for FLVCR2 in regulating heme homeostasis in vivo, it is interesting to note that Fowler syndrome, a vascular disorder of the brain that is caused by mutations in FLVCR2, is associated with defects in the activity of heme-containing respiratory complexes [192]. 

Another mechanism of heme import is via the uptake of hemoproteins. Haptoglobin and hemopexin, a pair of serum heme-scavenging proteins, bind to hemoglobin and heme, respectively released during hemolysis or cellular injury. Haptoglobin, which is secreted from hepatocytes, binds plasma hemoglobin with high affinity, *K*_D_ ≈ 10^−12^M [193] and this complex is endocytosed by macrophages via the receptor CD163 [194]. The heme from hemoglobin is subsequently released during endosomal acidification and imported into the cytoplasm and recycled by macrophages [195]. Hemopexin, which is primarily produced by the liver, binds “free” heme in the plasma with high affinity, *K*_D_ ≈ 10^−13^M [196] and this complex is endocytosed via the LRP1/CD91 receptor [196,197,198]. While LRP1 clearly plays a role in heme catabolism in the liver, its presence and role in cells other than hepatocytes is unclear and suggests it may function in heme uptake for utilization or as a source of iron [198].

In addition to heme transporters and receptor-mediated endocytosis of hemoproteins, heme uptake may also be mediated by the endocytosis of heme-loaded microparticles derived from red blood cells [199,200,201,202]. Although typically associated with red blood cell storage lesions or sickle-cell disease, circulating erythrocytes can release heme-laden microparticles that are highly inflammatory. These particles are endocytosed by endothelial cells in a Rab5-dependent manner [203] and constitute an additional mechanism of heme uptake by cells.

Another mechanism for the uptake of extracellular heme in mammals involves its sequestration by lipoprotein particles that can subsequently be endocytosed [101]. Free heme has the capacity to partition into low-density lipoproteins (LDLs) and high-density lipoproteins (HDLs) despite the presence of heme-scavenging proteins haptoglobin, hemopexin and albumin [101]. Once internalized, lipoproteins can release heme from acidic endosomal compartments. Altogether, multiple mechanisms account for the cellular import of exogenous heme for utilization or catabolism. Pathways for the endocytosis of exogenous heme have been identified in yeast as well, including budding and fission yeast, *S. cerevisiase* and *S. pombe*, respectively. In *S. cerevisiase*, FECH-depleted *hem15*∆ cells can be rescued by heme in fermentable media but not in non-fermentable media while cells are respiring [204,205]. The mechanism by which yeast cells import exogenous heme and the basis for why mitochondrial heme import is inefficient is not completely clear. A recent study identified gain-of-function mutant variants of the plasma membrane protein Nce102 as a genetic suppressor of respiratory defects in heme-supplemented *hem15*Δ mutants [83]. While the exact suppression mechanism remains elusive, the eisosome complex-related Nce102 [206] is likely to facilitate the uptake of exogenous heme via the endocytic pathway, wherein direct short-lived interactions between endosome and mitochondria mediate heme transfer between these subcellular compartments. In *S. pombe*, exogenous heme is endocytosed and trafficked to the vacuole for storage in a manner dependent on internalization by Shu1, a cell surface receptor and vacuolar transport by the ESCRT pathway [207,208,209,210].

### 4.3. Exogenous vs. Endogenous Heme

A long-standing dogma in heme cell biology field is that the cellular heme quota is solely dictated by de novo synthesis and degradation; any imported exogenous heme is thought to be degraded by heme oxygenases (HO) and the released iron is siphoned for de novo heme synthesis, stored, or utilized for any number of other iron-dependent processes. However, two major challenges to this paradigm are that: (i) heme supplementation rescues a number of cell and animal systems that are defective in heme synthesis, suggesting exogenous heme may in fact be recycled and reused independently of heme degradation [187,211,212]; and (ii) heme exporters [13,14] and inter-cellular heme chaperones [213] have been identified, which strongly suggests that heme can be shared between cells. Taken together, these new findings have prompted us to re-think the traditional paradigm that all heme for use in metabolism is derived from de novo synthesis in the mitochondria. However, the relative contributions of endogenously synthesized heme and exogenously supplied heme to cellular heme pools need to be determined. Further, there is little understanding of how imported heme is trafficked for degradation by HO in the ER or how cells bifurcate the flow of exogenous heme between HO and other heme-dependent processes.

A wealth of data indicates exogenous heme may be utilized in toto independently of its degradation. In the unicellular model eukaryote, yeast *S. cerevisiae*, *hem1*∆ cells, lacking the first enzyme in the heme synthesis pathway, ALA synthase, can be rescued to some extent by heme supplementation [204,205]. Further, in humans, patients suffering from certain porphyrias, a class of disorders characterized by heme synthesis defects, can be treated with intravenous heme and heme-albumin, which rescues heme-dependent enzyme activities in various tissues [214]. However, it is unclear how imported heme is distributed for use in metabolism in a manner that bypasses HO or what the mechanisms of heme uptake are for the mitochondria and other subcellular compartments. Interestingly, using horseradish peroxidase activity-based heme reporters targeted to different subcellular compartments in HEK293 cells, it was determined that exogenous and endogenous heme is trafficked differently to various subcellular compartments [174]. However, the mechanisms underlying the hierarchical distribution of exogenous and endogenous heme to various cellular locales are unknown. 

What are the mechanisms underlying the mobilization of heme out of the cell and what is the rationale for such a process? FLVCR1a is a 12 TMD plasma membrane heme export protein of the major facilitator superfamily of transporters. FLVCR1 was first discovered as a receptor for feline leukemia virus (FeLV) and its involvement in heme homeostasis was established based upon the development of aplastic anemia due to a loss of erythroid progenitors upon FeLV infection. It was subsequently determined that FLVCR1a is a cytoplasmic heme exporter that protects developing erythroid cells from heme toxicity and susceptibility to anemia [14,176]. As mentioned earlier, it was later determined that an alternative transcriptional start-site located within the first intron of FLVCR1a results in a shorter transcript that produces a truncated 6 TMD isoform, FLVCR1b, which transports heme out of the mitochondria [175]. Further, another FeLV receptor, the homologous cell surface protein, FLVCR2, was implicated as a heme importer [192].

FLVCR1a is thought to primarily protect cells from heme toxicity. For instance, it is thought that FLVCR1a exports heme from macrophages during erythrophagocytosis of senescent red blood cells. In fact, FLVCR1a interacts with hemopexin, an extracellular heme scavenger and exports heme > 100-fold more efficiently in the presence of hemopexin [215]. Further, FLVCR1a protects developing red blood cells from heme toxicity by acting as a safety valve to export potentially cytotoxic heme. In addition to its role in protecting cells from heme toxicity, there may also be inter-cellular signaling functions for heme. For instance, as a pro-inflammatory signaling molecule, erythrocyte-derived heme can differentiate and activate macrophages for clearance of red blood cells [15]. 

In addition to FLVCR1a, another mechanism for cellular heme export involves MRP-5, an ABC transporter (Figure 4) [216]. Loss of MRP-5 leads to embryonic lethality in *C. elegans* that can be rescued by addition of exogenous heme. Further, *Mrp5* deficient mice have reduced heme levels in the secretory pathway of embryonic fibroblasts. However, *Mrp5^−^*^/*−*^ mice do not show overt heme-related phenotypes, possibly due to compensation by other ABC-transporters. Taken together, like FLVCR1a, MRP5 provides yet another conduit for heme export from the cell; though its precise physiological function is unclear. Yet another protein shown to be involved in heme export is ABCG2, also known as the breast cancer resistance protein (BRCP). ABCG2 is an ATP-binding cassette transporter which has been shown to transport heme, hemin and porphyrin in addition to a variety of medications [217,218,219,220,221]. ABCG2 is expressed by a variety of normal tissues including placenta, brain, small intestines, ovary, liver and hematopoetic stem cells, as well as in a number of cancer cells [222]. Recent studies have shown that ABCG2 exports heme to serum albumin and that it functions in cellular protection [217]. While these export proteins suggest a protective function by which heme or porphyrin that is overexpressed or accumulated is exported for catabolism, such a process seems wasteful and potentially dangerous to the organism.

Another route for heme mobilization from cells is through secreted hemoproteins. In *C. elegans*, HRG-3, a 8 kDa intercellular heme chaperone protein, binds and delivers maternal heme to developing oocytes [213]. These results suggest that heme can be transferred between tissues and cell types as a nutritional heme source. However, the degree to which this happens in higher animals during development is unclear. Altogether, the identification of heme export pathways and heme rescue of phenotypes associated with defects in heme synthesis suggests heme can be utilized for metabolism independently of heme catabolism pathways.

### 4.4. Heme Trafficking Factors

The factors that regulate heme bioavailability and distribution are poorly understood. Historically, biochemically driven approaches toward probing heme homeostatic mechanisms and defining the heme proteome have been instrumental. For instance, cellular heme-binding proteins (HBPs) have been identified on the basis of their interaction with heme-agarose or blue-sepharose. These HBPs include 22 kDa HBP, 23 kDa HBP, SOUL, glutathione-S-transferase, fatty acid-binding protein (FABP) and glyceraldehyde phosphate dehydrogenase (GAPDH) [2,3,223]. However, with the exception of GAPDH, it is not known what role these HBPs play in regulating heme homeostasis. GAPDH was not only found to regulate heme insertion into nitric oxide synthase in a NO-dependent manner [224], but also found to buffer cytosolic heme and regulate its availability to a nuclear heme regulated transcription factor [91,92].

## 5. Multi-Model Comparison of Eukaryotic Heme Homeostasis

All heme-requiring organisms occupy a position on the continuum between relying exclusively on de novo heme synthesis and heme uptake. Most eukaryotes, including mammalian cells, are in the middle of this continuum and can both import and synthesize heme efficiently [174]. As mentioned previously, organisms like *C. elegans*, rely exclusively on heme uptake from nutritional sources and cannot make their own heme due to the lack of a functioning heme biosynthetic pathway [187]. As an interesting aside, these nematodes retained a pseudo FECH protein, encoded by the gene *fechl-1* (accession code: K07G5.6), with 23% identity to mammalian FECH. While this protein is expressed, it lacks canonical FECH activity [187,225,226]. Several other organisms including the cattle tick *Rhipicephalus (Boophilus) microplus* [227] and the Atlantic salmon louse *Lepeophtheirus salmonis* [228] are heme auxotrophs as well. *S. cerevisiae*, on the other hand, is not efficient at importing heme and is almost exclusively reliant on heme synthesis [211]. Since much of our current understanding of eukaryotic heme homeostasis comes from work in Baker’s yeast, mammalian cell lines and *C. elegans*, we have a window into common and divergent themes in heme homeostatic pathways of organisms that occupy different positions on the continuum of heme acquisition between synthesis and uptake (Table 1).

First, if an organism can make heme, eukaryotic heme synthesis is highly conserved and all eight heme biosynthetic enzymes share a high degree of homology across eukaryotic species. Second, all eukaryotes have the capacity to export heme, but with divergent mechanisms. For instance, Baker’s yeast expresses Pug1, a porphyrin-heme exchanger that imports PP IX while exporting heme [211]. On the other hand, in the model metazoan, *C. elegans*, intestinal heme is shared via a secreted hemoprotein, HRG3, that delivers heme to other tissues. In vertebrates, the heme transporter, FLVCR1a, is used to export heme into the extracellular space. On the basis for its conservation between worm and man, one mechanism that may be common for heme export is secretion via the secretory pathway, as proposed for MRP5-mediated heme export. Third, the import of heme is generally poorly defined across eukaryotes. While cell surface heme importers are known for *C. elegans*, e.g., HRG4, this factor is not well conserved in lower or higher eukaryotes. For instance, it is still not known what the high affinity heme importer is for mammalian cells or even if there is one. On the other hand, low affinity heme uptake factors like FLVCR2 and the folate transporter, HCP-1, have previously been identified in mammalian cells [3]. Fourth, the factors that mediate intracellular heme trafficking are largely unknown in any eukaryote. Two exceptions are GAPDH [91,92,224] and PGRMC1/2 [89,90], which are the only factors found to play roles in intracellular heme trafficking in both yeast and human cells lines, albeit their molecular mechanisms are not known. Overall, while heme synthesis is highly conserved across all of eukaryotic life, the transport and trafficking of heme is not and likely reflects the unique ecological niches that life adapted to in order to acquire heme and control its bioavailability.

## 6. New Methods to Probe Heme Trafficking

Total heme in the cell can be considered to be a sum of exchange-inert and exchange-labile pools [2]. Exchange-inert heme corresponds to heme buried in the active sites of high affinity hemoproteins, e.g., globins and cytochromes and is not readily exchangeable. On the other hand, exchange-labile heme can readily exchange between proteins and defines the bioavailable pool relevant to heme trafficking and signaling. Until recently, there were no methods to monitor bioavailable heme in intact living cells and most measurements simply reported “total” heme or the most highly absorbing and/or abundant hemoproteins (Table 2). As a highly colored pigment, historically, most measurements of heme from cell and tissue extracts involve the use of UV-visible absorbance spectroscopy. Indeed, heme exhibits Soret band extinction coefficients between ~30,000 cm^−1^ M^−1^ and ~200,000 cm^−1^ M^−1^ in the 390–420 nm spectral region, depending on oxidation state and the nature of coordinating protein ligands [136]. In samples with low turbidity, including body fluids like plasma, heme can readily be quantified from UV-vis spectroscopy. In addition, since the spectral characteristics of heme are sensitive to the identity of coordinating ligands and heme iron oxidation state, spectral deconvolution can resolve free non-proteinaceous heme from hemoglobin (Hb) [229]. Moreover, oxyHb and metHb can be readily differentiated as well [229]. However, due to light scattering in many biological matrices, including cells and tissues, heme is often extracted using strong acid, which serves to dissociate heme from proteinaceous ligands and organic solvents like acetone or butanol, which separates heme from the aqueous phase [230]. Extracted heme can be chromatographed by high performance liquid chromatography (HPLC) using a reverse-phase C18 column coupled to an in-line UV-vis detector for quantification [231,232]. This method allows for differentiating heme *b* from other heme types, including heme *o* or heme *a* [233]. Alternatively, extracted heme or heme from soluble lysates or isolated hemoproteins may be quantified using the pyridine hemochromagen assay, which is based upon the distinct spectral features of the bis-pyridine ferrous heme complex [234,235,236,237]. The development of commercial instrumentation to measure absorbing species in turbid samples, including in cells and tissues, has greatly facilitated the measurement of heme in biological matrices. The Olis CLARiTY UV-vis spectrophotometer employs a quartz cuvette within an integrating cavity absorption meter (ICAM) [237]. The quartz cuvette contains a reflective coating that effectively increases the path length, making the instrument much more sensitive to absorption of light in turbid samples [237,238]. Finally, the measurement of porphyrin fluorescence is a very sensitive technique for the quantification of heme, with a limit of detection of 10^−9^M [232,233]. While heme itself is non-fluorescent, de-metallation of the heme iron in boiling oxalic acid results in the formation of fluorescent PP IX free base (λ_ex_ = 400 nm; λ_em_ = 662 nm) [232,233]. 

Although the aforementioned methods serve as powerful tools for measuring heme, they suffer from a number of drawbacks. First, these methods often rely on cell and tissue disruption and homogenization, which is time consuming, technically demanding and may result in the loss of heme from the samples. Second, they do not provide information on heme distribution and localization, unless great care is taken to isolate organelles and subcompartments. Third, these methods do not resolve heterogeneity within populations of cells or the spatio-temporal dynamics of heme. Fourth, these methods fail to resolve the differences between total and bioavailable heme, the latter of which defines the pool of heme accessible for all heme-dependent functions.

Traditional methods to probe heme bioavailability involve assaying the activities of various heme-dependent enzymes and transcription factors in cell or tissue extracts, including cytochrome P_450_ enzymes (ER) [239,240], catalase (peroxisomes or mitochondria) [92,241], tryptophan 2,3 dioxygenase (cytosol) [242], indoleamine-2,3-dioxygenase (cytosol) [243], various nuclear transcription factors, e.g., Hap1 (in yeast), Bach1, p53 and Reverb-α/β [2,3,92,241] and peroxidases that can be genetically encoded and targeted to different subcellular compartments [174,244] (Table 2). While these methods have shed considerable insight into heme homeostatic mechanisms, they suffer from a number of drawbacks that arise from harsh lysis conditions and time-intensive enzyme assays. Therefore, these enzymatic assays may artifactually alter bioavailable heme pools due to the repartitioning of heme upon cell lysis and are unsuitable for probing the spatial and temporal dynamics of cellular heme due to the challenges associated with rapidly isolating organelles.

More recently, the development of genetically encoded fluorescent heme sensors has revolutionized the ability to image and probe labile heme relevant to its trafficking and signaling in intact living cells and subcellular compartments, circumventing the need for cell disruption and time-intensive enzyme assays. Independently and virtually contemporaneously, three labs reported the development of genetically encoded ratiometric fluorescent heme sensors. Song et al., [245] reported the first FRET sensor for cellular heme imaging. The sensor, CISDY, consists of a heme sensing moiety containing NEAT domains of a pair of heme transfer chaperones, IsdX1 and IsdC, tethered by a linker and flanked by enhanced cyan and yellow fluorescent proteins (ECFP and EYFP) at the N and C terminus, respectively. Heme binding induces the dimerization of IsdX1 and IsdC, resulting in an increase in the FRET efficiency between ECFP and EYFP. Expression of CISDY across various human cell lines and in locations spanning the cytosol, nucleus, mitochondria and ER, revealed labile heme to be ~20 nM in all these compartments. 

Following this development, Hanna et al., [92] reported the FRET-based heme sensor 1 (HS1), a fusion of cytochrome *b*_562_ (Cyt *b_562_*), enhanced green fluorescent protein (EGFP) and a red fluorescent protein, Katushka 2 (mKATE2). Heme binding to Cyt *b_562_*, a His/Met heme-binding protein from *E. coli* [246], results in > 90% quenching of EGFP fluorescence via FRET. mKATE2, on the other hand, is a poor FRET donor to heme [92] and its fluorescence is relatively insensitive to heme binding to the Cyt *b_562_* module. Thus, the ratio of heme-sensitive EGFP fluorescence to heme-insensitive mKATE2 fluorescence provides a readout of cellular heme. The application of HS1 has uncovered fundamental insight into heme trafficking, dynamics and signaling. For instance, the HS1 variant, HS1-M7A, was used to screen the yeast gene deletion collection to identify genes that regulate heme bioavailability. One factor that was identified to regulate heme bioavailability and trafficking to a nuclear heme-regulated transcription factor was the glycolytic enzyme, GAPDH [241]. Further, using HS1-M7A, it was found that subcellular heme pools are highly dynamic and can be mobilized by signaling molecules like nitric oxide (NO) [92] and heavy metal stress [241].

Even more recently, Abshire et al. [247] reported a FRET-type sensor consisting of the *Plasmodium falciparum* histidine-rich heme-binding protein 2 (*Pf*HRP2) flanked by ECFP and EYFP as FRET donor and acceptor proteins, respectively. Heme binding to *Pf*HRP2 results in a lowering of the fluorescence lifetime of ECFP due to energy transfer to the heme moiety and therefore a decrease in FRET between ECFP and EYFP. While the heme sensor domain non-cooperatively binds ~15–18 heme molecules/monomer with an apparent dissociation constant of ~0.3 µM, a “mini-library” of sensor variants results in sensors with varying heme-binding stoichiometries, ~5–15 and heme dissociation constants, 0.05–0.25 μM. Using these sensors in the malaria parasite, it was found that *P. falciparum* maintains the cytosolic labile heme pool at ~1.6 μM throughout its development in red blood cells. Further, it was found that anti-malarial drugs that target heme homeostatic mechanisms in *P. falciparum*, including chloroquine, significantly increase labile heme. Altogether, the advent of ratiometric fluorescent heme sensors has greatly expanded the tools available to probe heme homeostatic mechanisms and mobilization dynamics in health and disease in an unprecedented manner.

However, one potential drawback with the aforementioned heme sensors is that their expression may itself perturb heme homeostasis due to heme sequestration [2]. Moreover, different sensor scaffolds may access different heme pools due to kinetic constraints associated with heme exchange. Thus, measurements of labile or bioavailable heme may in effect be a sensor-dependent parameter. Due to these potential issues, measurements of bioavailable heme with heme sensors can be complemented by label-free heme imaging modalities that exploit the rich electronic and vibrational transitions of metalloporphyrins, including resonance Raman, photothermal lens and transient absorption microscopies (Table 2). Resonance Raman is a vibrational spectroscopy in which incident radiation from a laser that is resonant with an electronic transition of a target molecule is inelastically scattered upon excitation of various vibrational transitions [248]. The difference in energy of the incident radiation and the scattered photons is diagnostic of the specific molecules being excited and their local environment. In particular, resonance Raman has been used for decades to characterize the coordination environment of heme in various hemoproteins. In resonance Raman microscopy, resonance Raman spectroscopy is coupled with microscope optics to generate a “Raman map” that gives structural and chemical information with spatial resolution [249]. This method has been utilized to probe the activity of a number of hemoproteins in living cells, including NADPH oxidase activity in neutrophils and hemoglobin oxygenation in erythrocytes. Moreover, it was used to probe hemoglobin and hemozoin distribution during erythrocyte infection with *P. falciparum* [250]. 

Photothermal microscopy relies on the detection of local heating generated by optical absorption of molecules. When combined with two-photon excitation using longer wavelength light to excite a chromophore rather than one-photon linear absorption, there is a decrease in scatter, background signal and photo-bleaching with increased sample penetration depth. Heme is an ideal candidate for the use of two-photon photothermal lens microscopy as it absorbs strongly and is not fluorescent [251,252]. Using photo-thermal lens microscopy, the distribution of hemoproteins, including globins and cytochromes, in cell and tissue samples was measured in situ. 

Transient absorption microscopy (TAM) utilizes ultra-fast pulsed lasers to measure the excited state dynamics and spatial distribution of chromophores [253]. A “pump” laser is used to excite an absorbing species and a “probe” laser is used to measure the excited state dynamics of that molecule. The excited state dynamics are diagnostic of particular chromophores and can reveal the chemical nature and environment of a molecule [254]. Transient absorption microscopy is efficient at quantifying and visualizing chromophores, especially those that have low quantum yields [255]. TAM has been utilized to observe heme in frozen heart tissues as well as heme distribution and dynamics in *C. elegans* [253,256].

The major advantage of these methods is that samples do not need to be derivatized or genetically altered and heme can be probed in situ, ensuring that heme homeostasis and cellular metabolism is not perturbed. However, a limitation is that the spectroscopic features being probed may be derived from a complex heterogeneous mixture of heme species, making the deconvolution and interpretation of signal changes challenging. On the other hand, in cell types where the signal is dominated by a few abundant and/or highly absorbing heme complexes, e.g., hemoglobin in red blood cells, the application of such label-free techniques may be simpler to interpret. Altogether, the new heme sensors and label-free imaging of heme provide a new set of tools to further define the exciting questions related to heme trafficking in living intact cells.

## 7. Concluding Remarks

Heme is a versatile biological molecule of paramount importance for cellular physiology. Because of its involvement in virtually all aspects of aerobic life and high relevance to human health and pathology, this fascinating metallo-organic compound and its complex metabolism have been extensively studied. In spite of over six decades of heme research, many gaps in our understanding of cellular heme metabolism still remain. For example, the mechanisms that heme *b* uses to shuttle away from FECH and to route to its sites of utilization across the cell are only beginning to emerge. Development of novel models, tools and methods to quantitatively study trafficking of heme and its precursors in vivo is highly promising and likely will continue gaining some exciting new insights into the mechanisms behind heme transport and distribution within eukaryotic cells. Deciphering how heme is routed within and outside mitochondria as well as regulatory aspects of these processes will provide answers to a number of outstanding biological questions and contribute towards greater understanding of highly prevalent heme-related pathologies.

## Figures and Tables

**Table 1:** Conservation of heme homeostatic factors between yeast, worm and man.

Heme Homeostatic Process	Enzyme	*Saccharomyces cerevisiae*	*Caenorhabditis elegans*	*Homo sapiens*
Heme Synthesis	5-aminolevulinic acid synthase	Hem1	✖	ALAS1/ALAS2
	Porphobilinogen synthase	Hem2	✖	PBGS
	Hydroxymethylbilane synthase	Hem3	✖	HMBS
	Uroporphyrinogen synthase	Hem4	✖	UROS
	Uroporphyrinogen decarboxylase	Hem12	✖	UROD
	Coproporphyrinogen oxidase	Hem13	✖	CPOX
	Protoporphyrinogen oxidase	Hem14	✖	PPOX
	Ferrochelatase	Hem15	fecl-1	FECH
Heme Degradation	Heme oxygenase	Hmx1	?	Hmox1/Hmox2
Heme Import	FLVCR2	✖	✔	✔
	HRG4	✖	✔	✖
Heme Export	FLVCR1	✖	✖	✔
	MRP5	✖	mrp-5	ABCC5
	Pug1	✔	✖	✖
	HRG3	✖	✔	✖
Heme Trafficking	PGRMC1/2	Dap1	vem-1	PGRMC1/2
	GAPDH	Tdh1/2/3	gpd1/2/3/4	GAPDH
	HRG1	✖	✔	✔

**Table 2:** Approaches to measure total and labile heme in cells and tissues. References are in the text.

Approaches	Methods	Advantages	Disadvantages
In Situ Label Free Imaging	Transient Absorption Microscopy Resonance Raman Imaging 2 Photon Photothermal Lens Microscopy	Subcellular resolution (<1 μm) Non-invasive Can probe heme dynamics in living cells	Signals dominated by most abundant and/or highly absorbing species Low-throughput Requires specialized equipment/expertise
In Situ Imaging of Labile Heme using Fluorescent Heme Sensors	HS1 CISDY-9 CHY	Subcellular resolution (<1 μm) Direct probe of labile “bioavailable” heme Can probe heme dynamics in living cells High-throughput	May perturb heme homeostasis Possible selection bias depending on the nature of the sensor Extended time resolved studies precluded by photobleaching
Assays for Endogenous Markers of Heme Bioavailability	Horseradish Peroxidase Tryptophan 2,3 Dioxygenase (TDO) Indoleamine-2,3-Dioxygenase (IDO) Cytochrome P450 Catalase Transcription Factors	Measurement of heme accessible to endogenous hemoproteins No genetic perturbations	Disruption of cells and tissues Time consuming Difficult to get fast time resolution
Assays for Total Heme	HPLC	Resolve different heme types	Time consuming Disruption of cells and tissues Low-throughput
	Porphyrin Fluorescence	nM sensitivity High-throughput	Disruption of cells and tissues
UV/vis Absorbance Spectroscopy	CLARiTY	Sensitive measurements in turbid samples Possible to measure heme and hemoproteins in intact cells	Signals dominated by most abundant and/or highly absorbing species Low-throughput Requires specialized equipment
	Pyridine Hemochromagen	Broadly accessible Inexpensive	Disruption of cells and tissues

## Abbreviations

CO	Carbon monoxide
IMS	Mitochondrial intermembrane space
TCA	Tricarboxic acid
IMM	Inner mitochondrial membrane
5-ALA	5-aminolevulunic acid
ALAS	Aminolevulunic acid synthase
PBGS	Porphobilinogen synthase
PBG	Porphobilinogen
HMB	Hydroxymethylbilane
HMBS	Hydroxymethylbilane synthase
UPgen III	Uroporphyrinogen III
UROS	Uroporphyrinogen synthase
CPgen III	Coproporphyrinogen III
UROD	Uroporphyrinogen decarboxylase
PPgen IX	Protoporphyrinogen IX
CPOX	Coproporphyrinogen oxidase
Fe-PPIX	Iron-protoporphyrin IX
PPIX	Protoporphyrin IX
PPOX	Protoporphyrinogen oxidase
FECH	Ferrochelatase
PLP	Pyridoxal 5’-phosphate
OMM	Outer mitochondrial membrane
SLC	Solute carrier
α-KG	A-ketoglutarate
MTS	Mitochondria-targeting sequence
HRM	Heme regulatory motif
IRP	Iron regulatory protein
ATP	Adenosine triphosphate
ADP	Adenosine diphosphate
AAA+	ATP hydrolase associate with various cellular activities
ANT	Adenine nucleotide translocator
KDH	A-ketoglutarate dehydrogenase
CcO	Cytochrome *c* oxidase
ETC	Electron transport chain
SDH	Succinate dehydrogenase
CCHL	Cytochrome *c* heme lyase
HCCS	Holocytochrome *c* synthase
MLS	Microphthalmia with linear skin defects
TMD	Transmembrane domain
FDX	Ferredoxin
MAMs	Mitochondria-associated membranes
ERMES	Endoplasmic reticulum-mitochondria encounter structure
MDV	Mitochondrial-derived vesicle
HRG	Heme responsive gene
ZnMP	Zinc mesoporphyrin
CHO	Chinese hamster ovary
LDL	Low-density lipoprotein
HDL	High-density lipoprotein
HO	Heme oxygenase
FeLV	Feline leukemia virus
ABC	ATP-binding cassette
BRCP	Breast cancer resistance protein
HBP	Heme-binding protein
FABP	Fatty acid-binding protein
GAPDH	Glyceraldehyde phosphate dehydrogenase
PGRMC1/2	Progesterone receptor membrane component 1/2
EGFP	Enhanced green fluorescent protein
ECFP	Enhanced cyan fluorescent protein
EYFP	Enhanced yellow fluorescent protein
HS	Heme sensor
FRET	Forster resonance energy transfer
